# Systems medicine: total body positron emission tomography meets omics

**DOI:** 10.1093/bjr/tqag050

**Published:** 2026-02-26

**Authors:** Angus K Jacobs, Athena Chan, Adriana A S Tavares

**Affiliations:** Institute for Neuroscience and Cardiovascular Research, University of Edinburgh, Edinburgh, EH16 4TJ, United Kingdom; Institute for Neuroscience and Cardiovascular Research, University of Edinburgh, Edinburgh, EH16 4TJ, United Kingdom; Institute for Neuroscience and Cardiovascular Research, University of Edinburgh, Edinburgh, EH16 4TJ, United Kingdom; Edinburgh Imaging, University of Edinburgh, Edinburgh, EH16 4TJ, United Kingdom

**Keywords:** total-body PET, omics imaging, proteomics, genomics, metabolomics, connectome analysis, pathway analysis

## Abstract

Omics imaging has emerged as an interdisciplinary field focused on integrating data collected from biomedical images and omics analysis. Historically, there is precedent for omics imaging to serve as a superior model for prediction of human diseases, including brain disorders and cancer, versus single technique models (eg, only imaging data or only omics data). Most of the previous studies in the field of omics imaging have focused on single organ analysis. However, the advent of total-body positron emission tomography (PET) imaging for clinical use has the potential to transform this landscape by enabling high sensitivity or high throughput multi-organ analysis with methodologies previously established for analysis of omics datasets, such as connectome and pathway analysis tools. Conversely, traditional omics analysis, which lack spatial and structural multi-organ information, can benefit from total-body PET imaging of molecular targets *in vivo* across multiple organs in humans. In this commentary, the importance of whole-person research enabled by total-body PET, integration of total-body PET with omics techniques and examples of successful case studies of imaging omics are described. Although the field of imaging omics is relatively new, discoveries already enabled by this field provide seminal evidence of its importance to advance human disease diagnosis, prognosis and treatment.

## Introduction

The concepts of global disease assessment and systems medicine are common place in the omics field, where genomics, proteomics, and metabolomics have provided a multidimensional evaluation of disease heterogeneity and systemic effects *in vivo.*^1^ This has led to a more complete understanding of how diseases affect the body.

While these classical omics approaches provide detailed functional and pathway-related insight at the tissue or cellular level, they typically offer limited spatial or structural insight across multiple organs, which is essential for understanding disease progression at the systems-level. There has been a lot of recent interest surrounding combining classical omics-based approaches with multi-organ molecular imaging modalities. From inception, positron emission tomography (PET) imaging has been at the forefront of molecular imaging techniques^2^ and the recent development of total-body PET technology has the potential to transform systems medicine.^3^ This can be realized by exploiting PET selective molecular targeting via radiotracers and total-body PET maximized sensitivity with whole-body coverage. As described by Sundar *et al*, total-body PET has the potential to be a catalyst for whole-person research by enabling multi-variate biosignal analysis and visualizing interactions between organs.^4^ This could in turn be used to generate new systems biology hypotheses for subsequent translational studies across scales, from cells to whole-body.

Here, we discuss how some of the recent advances in long-axial field of view (LAFOV) PET have augmented radiomics-based approaches; how classical omics technologies have been utilized for PET tracer development and their applications; and the future potential opportunities for integrating omics and LAFOV PET.

## LAFOV PET and radiomics

The term omics imaging has emerged as an interdisciplinary field focused on integrating data collected from biomedical images and omics analysis.^5^ There are already several examples where this integration has been successful and yielded important new knowledge. For example, studies have shown that genetic variations are associated with human brain volume differences.^6^ Another example stems from Parkinson’s disease research where a linear regression model combining genetic and neuroimaging features predicted the Movement Disorder Society-sponsored unified Parkinson’s disease rating scale (MDS-UPDRS) compared to other models using only genetic or neuroimaging information alone.^7^ This combinatorial gain is likely to expand beyond single organ applications and LAFOV PET provides a unique opportunity window to investigate this across multiple systems in the whole body.

Recently published studies have started to probe the new systems medicine paradigm using LAFOV PET data and connectome analysis.^8^^,^^9^ The application of traditional omics methodology, such as connectome analysis, to mine LAFOV PET datasets can deliver transformational insight into how human diseases develop, progress and respond to treatment at a multi-system scale. For example, recently, we have shown that in lung cancer patients without metastatic disease, [^18^F]FDG skeletal networks prior to start of treatment are significant predictors of patient survival and this methodology performs better than established primary tumour standardised uptake value (SUV) threshold analysis.^8^ This illustrates that LAFOV PET analysis beyond SUV can be powerful tools for clinical management of patients. Other studies have also identified important molecular organ axes, such as the heart: brain axis following myocardial infarction using 18 kDa translocator protein (TSPO) PET ligands.^10^^,^^11^ Collectively, these seminal studies have supported the view that systems medicine approaches are needed to deliver state-of-the-art personalized medical care. Although the route to deploy network and connectome LAFOV PET techniques in the clinical routine space will require translation and packaging of research techniques for mainstream clinical use, the potential is there, for both established radiotracers like [^18^F]FDG and new radiotracers.

Pathway analysis is a complementary approach with particular promise in LAFOV PET. Although pathway and network analyses are well-established in brain PET imaging for mapping neural circuits (eg,^12^) they have not yet been thoroughly explored in LAFOV PET. Crucially, this pathway-centric perspective is bidirectional: not only can PET imaging benefit from repurposing omics-type analytical tools, but the reverse is also true. The powerful nature of large-scale omics datasets often lacks spatial coordination with different organs and regional tissue changes. This limitation could be overcome by combining omics screening with LAFOV PET analysis, so the changes identified using genomics, transcriptomics, proteomics and metabolomics in humans can be spatially mapped into multi-organ systems *in vivo*. This approach could be leveraged by making use of established and successful large omics projects, such as the human genome project,^13^ whereby genetic profiles could be matched to whole-body multi-organ profiles.

## Integrating multi-omics technologies in radiotracer development

In the context of new radiotracer development, omics techniques can play a central role in target identification. A recent example of a data-driven multi-omics approach for the identification of novel radiopharmaceutical targets has been in pancreatic ductal adenocarcinoma (PDAC). Wang *et al*, highlighted claudin-4 as a suitable cancer cell marker using spatial transcriptomics and proteomics of human biopsies, characterizing the localization and a ∼16-fold increase in cancer compared with healthy pancreas.^14^ They went on to develop a peptide-based claudin-4 PET imaging probe that appeared to show clear uptake in a preclinical PDAC model. Though this is the first multi-omics approach integrating spatial transcriptomics for identifying and characterizing a novel PET radiopharmaceutical target, as the cost and availability of multi-omics technologies improves, there is clear scope for utilizing multi-omics approaches for molecular imaging target identification.

Furthermore, through use of pathway analysis, another classic omics technique, LAFOV PET imaging data could be used to rapidly identify multiple applications for new radiotracers outside of the originally targeted organ system. The concept of pathway analysis is not new in brain PET imaging, where network analysis has long been used for mapping brain pathways (eg,^12^) but hasn’t yet been thoroughly explored in whole-body PET imaging. As such, PET radiotracer development can beneficiate from the repurposing of omics-type analysis tools.

## Future application of LAFOV PET and omics

Looking ahead, the use of multi-omics approaches, namely, genomics, transcriptomics, proteomics, metabolomics, and radiomics, could change how we study and understand phenotypic changes in human cells and organs–allowing us to describe and model disease characteristics better, in order to accelerate the treatment of human diseases.^15^ For example, disease-associated transcriptomic or proteomic changes can be correlated with patient phenotypic PET imaging data, with epigenetics analysis used to understand the impact of pathogenic factors and comorbidities in the development and progression of disease. This holistic approach could provide a link between genetics background and ultimate phenotypic changes via epigenetics analysis.^16^

## Conclusions

**Figure 1.**
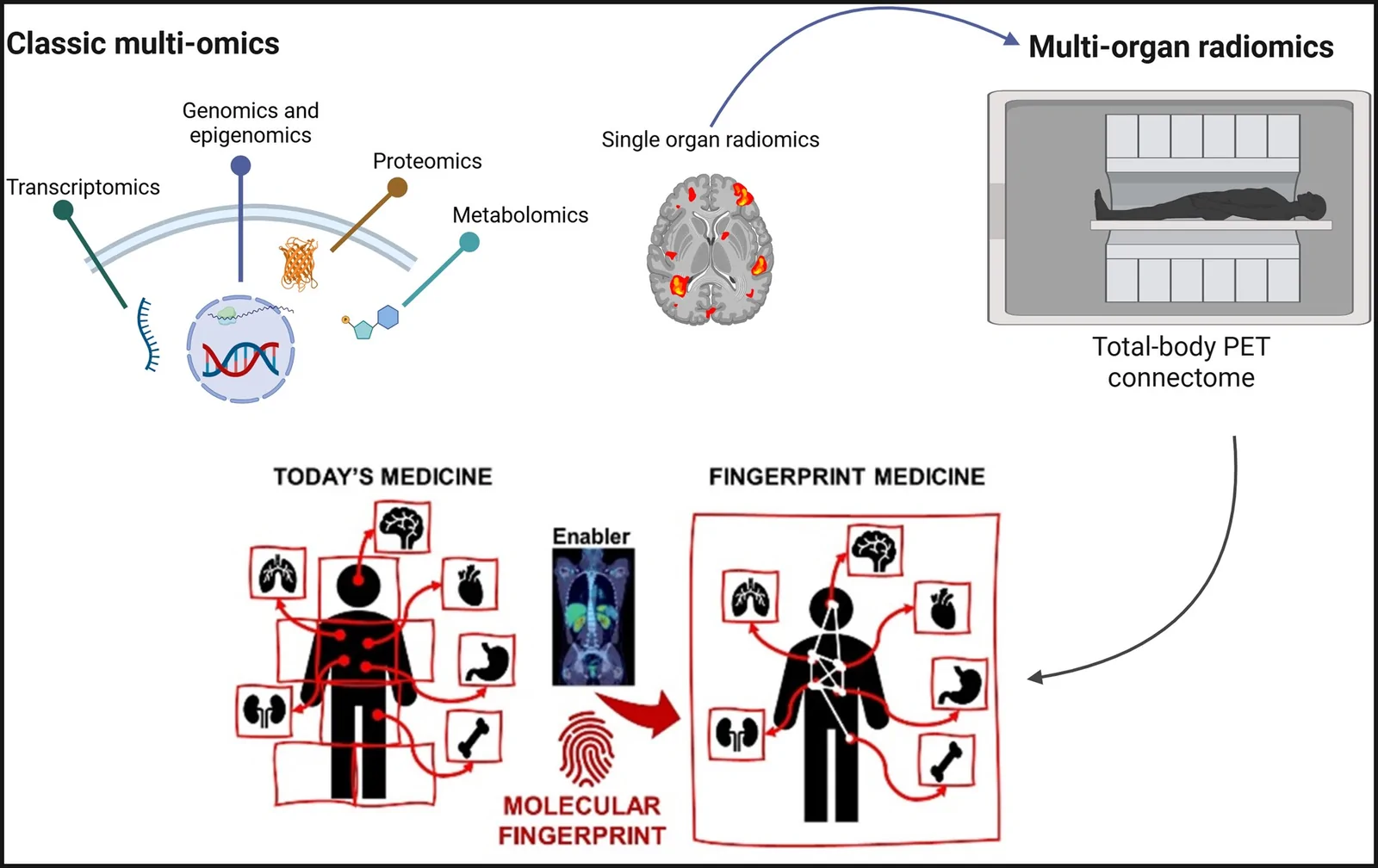
Total-body PET adds a dimension to classic single organ radiomics by enabling whole-body radiomics. Combined with genomics, transcriptomics, proteomics and metabolomics, total-body PET radiomics (eg, connectome analysis and molecular fingerprinting) can deliver personalized systems medicine.

In conclusion, the recent technological development of total-body PET imaging is poised to catapult whole-person discoveries in the coming years (Figure 1). Its significant potential to deliver on imaging omics is a perfect match to genomics, transcriptomics, proteomics and metabolomics. The meeting of these 2 worlds has already provided exciting new insights as well as symbiotic cross-pollination of methodologies, while improving disease predictive models of superior quality than those developed using each tool separately.
